# Complete genome sequence of bacteriophage MrAaronian isolated from an *Arthrobacter globiformis* culture

**DOI:** 10.1128/MRA.00778-23

**Published:** 2023-11-07

**Authors:** Ian Haines, Jessica Blakely, Ashley Branson, Diana Estrada, Rebeca Fernandez Robles, Katelyn Fitzgerald, Shelby Jeffers, Timyee Leung, Jasmine Munoz, Ashley Olivos, Anayeli Ramirez, Caressa Smith, Justin Spere, Idaleth Tavarez, Isabella Wood, Ethan Zavala, Madison Arrighi, Angelica Coronel-Galindo, Megan K. Dennis, Marlee Goppert, Dustin Edwards

**Affiliations:** 1 Department of Biological Sciences, Tarleton State University, Stephenville, Texas, USA; 2 Department of Biology, Marist College, Poughkeepsie, New York, USA; Department of Biology, Queens College, Queens, New York, USA

**Keywords:** bacteriophages, *Arthrobacter*

## Abstract

Arthrobacteriophage MrAaronian contains a 54,509 bp DNA genome with 87 predicted protein-coding genes. MrAaronian has siphovirus morphology and was collected from a flowerbed soil sample in Poughkeepsie, NY, and isolated on an *Arthrobacter globiformis* B-2979 culture. MrAaronian has > 99% nucleotide identity with cluster AW arthrobacteriophages Michelle, Stayer, Sloopyjoe, and StarLord.

## ANNOUNCEMENT

The *Arthrobacter* group of bacteria is unusual since they appear as Gram-negative rods in young cultures and as Gram-positive cocci in older cultures (1, 2). *Arthrobacter globiformis*, a soil-dwelling bacterium with potential commercial importance, is able to degrade several pollutants, including hexavalent chromium (3). Further characterization of bacteriophages and viruses that infect specific host bacteria, targeting *Arthrobacter* could have applications in selective bioremediation.

**Fig 1:**
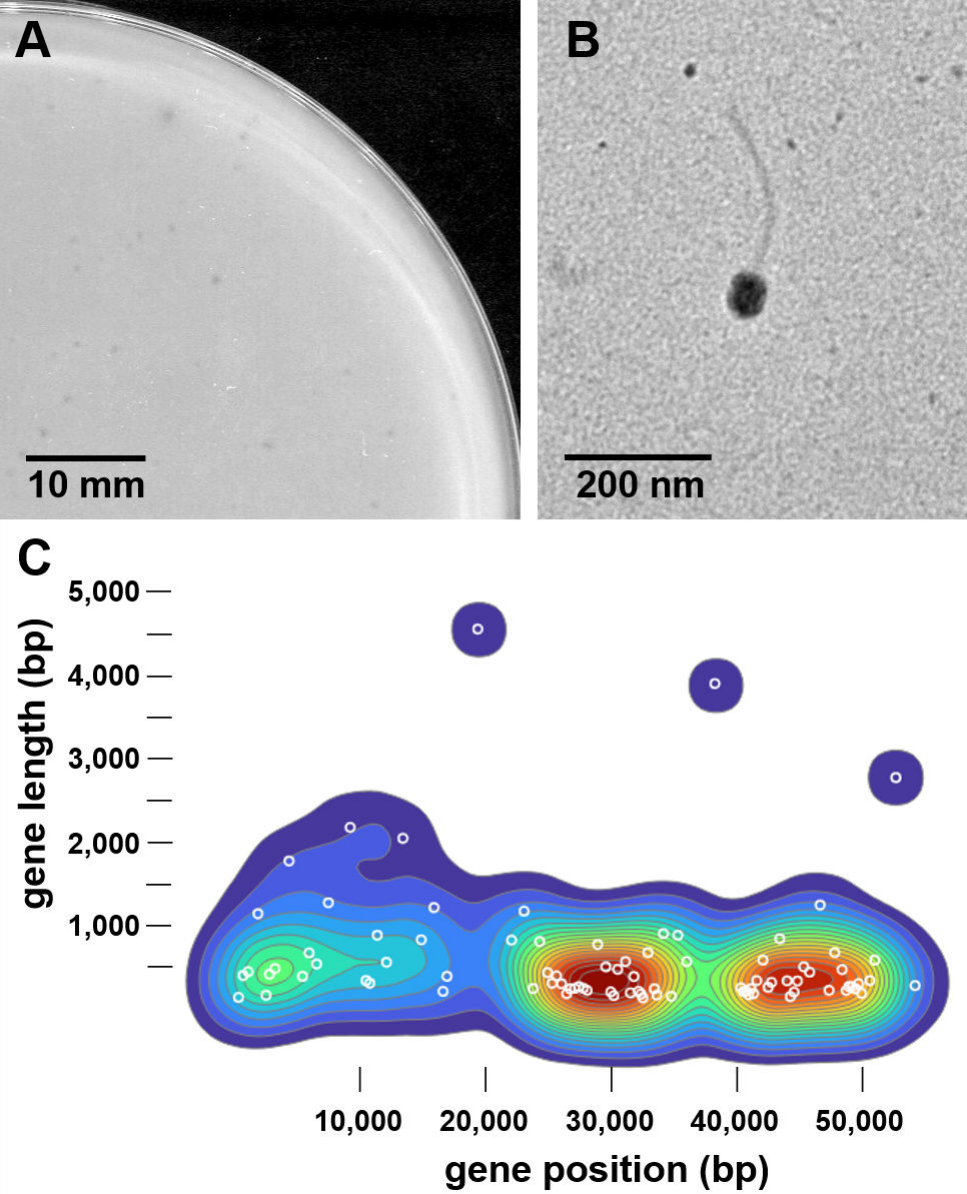
Characterization of arthrobacteriophage MrAaronian. (**A**) Petri dish (90 mm) containing PYCa solid culture medium and bacterial host *Arthrobacter globiformis* B-2979 infected with MrAaronian. After incubation for 24 h at 30°C, plaques appeared to be <1 mm in diameter and clear, with no visible halo (scale bar = 10 mm). (**B**) Transmission electron micrograph of MrAaronian high-titer lysate negatively stained with 1% uranyl acetate on a 300-mesh copper grid and imaged with a Hitachi HT7800 120 kV transmission electron microscope. MrAaronian has a siphovirus morphology with an approximate tail length of 285 nm and a capsid diameter of 67.5 nm (scale bar = 200 nm). (**C**) A density plot showing the relationship between length and position of genes in cluster AW bacteriophages and MrAaronian (white circles).

Arthrobacteriophage MrAaronian was isolated from a moist soil sample in Poughkeepsie, NY [global positioning system (GPS) coordinates: 41.72201 N, 73.930158 W]. The sample was washed with peptone-yeast calcium (PYCa) liquid media, and the supernatant was passed through a 0.22 µm syringe filter. The filtrate was incubated with host *A. globiformis* B-2979 in a soft agar overlay on PYCa agar plates at 30°C. Bacteriophages forming small, lytic plaques were isolated by two rounds of picking a single, well-separated plaque, followed by diluting the bacteriophage sample in a 10-fold dilution series and plating again with *A. globiformis* (Fig. 1A). High-titer lysates were prepared by flooding “webbed” plates with phage buffer [10  mM Tris (pH 7.5), 10  mM MgSO4, 68  mM NaCl, 1  mM CaCl2, 10% glycerol], as described in the *Phage Discovery Guide* (4). Negative-staining transmission electron microscopy showed a siphovirus morphology, and ImageJ v1.53m (5) measured an approximate tail length of 285 nm and capsid diameter of 67.5 nm (Fig. 1B).

Bacteriophage genomic DNA was extracted using a Promega Wizard DNA Clean-Up System (Promega, Madison, WI), and sequencing libraries were prepared using the NEBNext Ultra II DNA Library Prep Kit (New England Biolabs, Ipswich, MA). Libraries were sequenced by Illumina MiSeq at the Pittsburgh Bacteriophage Institute to generate 607,351 total single-end reads of 150-base read length. A single bacteriophage contig with 6,670× coverage was assembled using Newbler v2.9 and checked for completeness and genomic termini using Consed v29 (6, 7). The 54,509 bp double-stranded DNA genome contains a 9-nucleotide 3′ single-stranded overhang (5′-CGCCGACCT- 3’) and 51.7% G + C content.

BLASTn (8) query of the sequence with the nonredundant/nucleotide (nr/nt) database returned >99% nucleotide sequence identity to cluster AW bacteriophages Michelle, Stayer, Sloopyjoe, and StarLoad (Table 1). Auto-annotations by GLIMMER v3.02 (9) and GeneMark v2.5p (10) were manually refined using Phamerator (11), PECAAN (https://discover.kbrinsgd.org), and DNA Master v5.23.6 (http://phagesdb.org/DNAMaster/). Consistent with other cluster AW bacteriophages, no tRNA genes were identified using Aragorn v1.1 (12) and tRNAscan-SE v2.0 (13). TmHmm and SOSUI v1.11 (14) assessed potential trans-membrane helices for each gene product. The genome contained rightward transcribing genes and putative functions was assigned to 25 of 87 protein-coding genes using BLASTp v2.13 (8) and HHPred v3.3 (15), which include structural proteins, endolysin, DNA primase/polymerase, DNA helicase, ssDNA and dsDNA binding proteins, four membrane proteins, two HNH endonucleases, VRR-Nuc domain protein, and Cas4 exonuclease. Gene position and length of MrAaronian were compared against other cluster AW members in a density plot using observable (16) showing three gene groupings (Fig. 1C). All tools were run with default parameters.

**TABLE 1:** Characteristics of similar^*a*^ arthrobacteriophages in cluster AW

Phage name	Length of genome (bp)	G+C content (%)	GenBank accession number	Similarity to MrAaronian (%)
MrAaronian	54,509	51.7	OR159662	
Michelle	54,509	51.7	MN234210	99.71
Stayer	54,507	51.7	MN234175	99.37
Sloopyjoe	54,511	51.7	OP910131	99.24
StarLord	54,504	51.7	MN234229	99.15

^*a*^ Similar being defined as having a nucleotide similarity over 95%.

## Data Availability

Actinobacteriophage MrAaronian genome is available in GenBank as accession number OR159662. Raw reads are available in the SRA under accession number SRX21368075.
